# Multiple Browsers Structure Tree Recruitment in Logged Temperate Forests

**DOI:** 10.1371/journal.pone.0166783

**Published:** 2016-11-28

**Authors:** Edward K. Faison, Stephen DeStefano, David R. Foster, Joshua M. Rapp, Justin A. Compton

**Affiliations:** 1 Highstead Foundation, Redding, Connecticut, United States of America; 2 U. S. Geological Survey, Massachusetts Cooperative Fish and Wildlife Research Unit, University of Massachusetts, Amherst, Massachusetts, United States of America; 3 Harvard Forest, Harvard University, Petersham, Massachusetts, United States of America; 4 Springfield College, Springfield, Massachusetts, United States of America; Lakehead University, CANADA

## Abstract

Historical extirpations have resulted in depauperate large herbivore assemblages in many northern forests. In eastern North America, most forests are inhabited by a single wild ungulate species, white-tailed deer (*Odocoileus virginianus)*, and relationships between deer densities and impacts on forest regeneration are correspondingly well documented. Recent recolonizations by moose (*Alces americanus*) in northeastern regions complicate established deer density thresholds and predictions of browsing impacts on forest dynamics because size and foraging differences between the two animals suggest a lack of functional redundancy. We asked to what extent low densities of deer + moose would structure forest communities differently from that of low densities of deer in recently logged patch cuts of Massachusetts, USA. In each site, a randomized block with three treatment levels of large herbivores–no-ungulates (full exclosure), deer (partial exclosure), and deer + moose (control) was established. After 6–7 years, deer + moose reduced stem densities and basal area by 2-3-fold, *Prunus pensylvanica* and *Quercus* spp. recruitment by 3–6 fold, and species richness by 1.7 species (19%). In contrast, in the partial exclosures, deer had non-significant effects on stem density, basal area, and species composition, but significantly reduced species richness by 2.5 species on average (28%). Deer browsing in the partial exclosure was more selective than deer + moose browsing together, perhaps contributing to the decline in species richness in the former treatment and the lack of additional decline in the latter. Moose used the control plots at roughly the same frequency as deer (as determined by remote camera traps), suggesting that the much larger moose was the dominant browser species in terms of animal biomass in these cuts. A lack of functional redundancy with respect to foraging behavior between sympatric large herbivores may explain combined browsing effects that were both large and complex.

## Introduction

With the widespread loss of large herbivores at the end of the Pleistocene and in the past several hundred years, much of our accumulated understanding of temperate forest dynamics comes from systems with depauperate large herbivore assemblages [1–3]. In the eastern deciduous forests of North America, intensive hunting during the nineteenth century resulted in the extirpations of bison (*Bison bison*), elk (*Cervus canadensis*), and moose (*Alces americanus*), leaving a species-poor native ungulate fauna in the twentieth century generally dominated by a single herbivore (white-tailed deer; *Odocoileus virginianus*) [4]. One of the research outcomes of this loss of species diversity has been the intensive study of a single ungulate’s impact on temperate forest dynamics, producing generally well documented relationships between white-tailed deer densities and vegetation impacts in a particular habitat context [5,6]. For example, in forest openings located in densely forested landscapes, deer densities below about 6–7 deer km^-2^ are generally compatible with vigorous and diverse tree recruitment and herbaceous layers [6–9]. At densities ≥8 deer km^-2^, tree and shrub density and richness begin to decline notably ([7,8,10,11]. In mature forest preserves surrounded by agricultural fields, abundant supplemental forage buffers forest understories against deer herbivory up to densities of 20 km^-2^[12]. In addition, variation in forage density within similar habitats influences the extent to which a given density of deer will impact a site [13].

Identifying density thresholds for more than one ungulate species is considerably more difficult and complex, given the diversity of foraging strategies, diets, and impacts of different-sized herbivores that result in little functional redundancy among species [14,15]. Consequently little is known about how multiple ungulate species at varying densities influence plant communities [16]. This uncertainty presents a challenge in areas where ungulate species have recently recolonized their former range or are predicted to expand their range with climate change, because it is unclear how the arrival of the new herbivore will alter known ungulate density-forest relationships in those systems or perhaps modulate predicted changes to plant communities with climate change [17]. In addition to different feeding strategies and foraging reach of the animals [15,18], a larger ungulate species may facilitate foraging opportunities for the existing smaller one by reducing the height of vegetation and creating more abundant and palatable lower shoots [1,19]. Moreover, the addition of a new herbivore could have either additive (compounding) or compensatory (opposing) effects on the vegetation depending on whether the same or different plant species are consumed respectively [20,21]. Compensatory effects can occur if two herbivore species consume primarily different plant species, thereby distributing consumption more evenly across the plant community and resulting in little net effect on relative species abundances. [20].

In the late 20^th^ century, moose (*Alces americanus*) recolonized much of their former southern range in the northeastern deciduous forest region (Massachusetts and northern Connecticut), creating a two-ungulate system with white-tailed deer in Massachusetts for the first time in about 200 years [22,23]. Estimated densities of white-tailed deer in central Massachusetts (3.8–5.8 km^-2^)[24,25] fall below the threshold for negatively impacting forest regeneration [5,8]. Estimated moose densities (0.2 km^-2^) are also generally low [23,26], suggesting their effects on forest regeneration could be relatively unimportant [14,27]. However, low ambient moose densities (e.g., 0.1–0.8 km^-2^) in undisturbed forests can increase dramatically (e.g., to 3.4–4.4 km^-2^) in nearby early successional stands disturbed by fire or logging [28], suggesting that impacts by moose could be large in these cuts despite their low ambient densities. In contrast, white-tailed deer use early successional and mature forests more evenly than do moose [29,30,31].

Adding to the lack of functional redundancy between deer and moose are the diet and size differences of the two animals: moose are 90% browsers compared to white-tailed deer, which are more mixed feeders (60% browsers, 40% grazers; [18]). Moose are also ~7 times the mass of deer [32], forage on woody plants 1–1.5 meters taller than those browsed by deer, and can pull down and snap small trees up to 4–5 cm in diameter to browse the leading shoots [22,33], much the way elephants (*Loxodonta africana*) do in tropical forests of Africa [34]. Thus, the presence of moose greatly extends the “browser trap” that trees must pass through [3].

Here we explore the role of moose and white-tailed deer in structuring temperate forest communities following canopy removal disturbance. In conducting this experiment, we asked whether the impact of recolonizing moose on density, composition, and diversity of tree recruitment was proportionate to their low ambient densities–i.e., deer + moose would have little additional impact on forests compared to impacts caused by the more abundant deer? or whether the propensity for moose to attain disproportionately high local densities in recently disturbed forests would result in much greater effects by deer + moose relative to deer alone?

## Materials and Methods

The physiography of Central Massachusetts is characterized by rolling plateaus with hills, and the climate is temperate with warm summers and cold winters [35,36]. Mean annual precipitation ranges from 97–127 cm per year, and mean temperature ranges from -12 –-0.5°C in January and 14–28°C in July. Mature forest vegetation is characterized by transition hardwood forests–mixed *Quercus*, *Acer rubrum*, *Betula lenta*, and *Fagus grandifolia*–with significant components of *Tsuga canadensis* and *Pinus strobus* [37,38]. In addition to timber harvesting, exotic forest insects and pathogens, including hemlock woolly adelgid (*Adelges tsugae*), beech bark disease (*Cryptococcus fagisuga* and *Nectria* spp.), chestnut blight (*Cryphonectria parasitica*), and gypsy moths (*Lymantria dispar*); and meteorological events (ice and windstorms) are the prevalent disturbances in the region [37].

**Fig 1:**
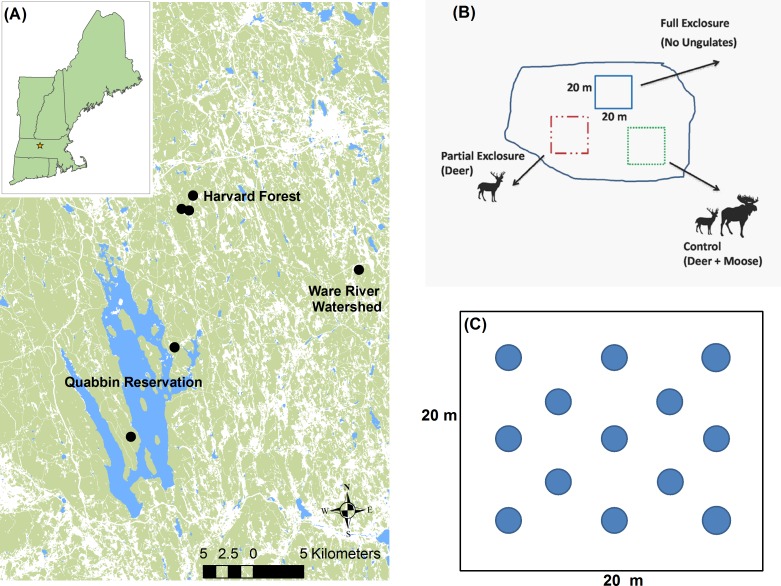
Map showing (A) location of study area and six study blocks in New England, USA; (B) experimental design showing three browser treatments; and (C) layout of 13 4-m^2^ circular subplots within each treatment plot.

In 2007–2008, six mixed conifer-hardwood stands (0.3–6 ha in size) that had been clearcut within the past 3–6 months at the Harvard Forest and the Quabbin and Ware River Watershed forests in Central Massachusetts were selected as study sites (Fig 1A and Table 1). David Foster, the third author on the paper, issued permission to use the Harvard Forest Sites; and Paul Lyons, Environmental Analyst, Massachusetts Department of Conservation and Recreation, Division of Water Supply Protection gave permission to use the Quabbin and Ware River sites. In each site, a randomized block with three treatment levels of large herbivores–no-ungulates (full exclosure), deer (partial exclosure), and deer + moose (control) was established (Fig 1B). A fourth treatment that excluded deer but was open to the larger moose was not feasible [20]. A number of recent large herbivore studies have utilized additive designs with semi-permeable exclosures similar to ours [15,39–41]. Blocks were at least 700 meters apart from one another. The 2.5-m tall exclosures were made of high-tension wire game fence with 15 cm grid mesh that enabled access to small mammals including lagomorphs and rodents. The full exclosure was fenced to the ground; the partial exclosure had a 60 cm opening between the bottom of the fence and the ground surface that excluded moose but allowed access to deer and all other wildlife; and the control plot was unfenced and open to both browsers (Fig 1B). Before erecting the fences, we consulted with several state wildlife biologists and the literature about an appropriate gap height at the bottom of the partial exclosure fences. Sixty centimeters was decided as the optimal height, which was more than twice the 25 cm reported in the literature as providing adequate passage for an adult white-tailed deer [42]. The Institutional Animal Care and Use Committee (IACUC) approved these methods and protocol.

**Table 1:** Characteristics of study site blocks.

Block	Location	Previous overstory composition	Dominant regeneration species	Opening size (ha)	Age of plot (yrs. since exclosures built)
Dana	Quabbin Reservation Forest	• *Quercus* • *Acer rubrum* • *Betula lenta*	• *A*. *rubrum* • *B*. *lenta* • *P*. *strobus*	0.4	6.7
Fisher	Harvard Forest	• *Pinus resinosa* • *P*. *strobus* • *B*. *lenta* • *A*. *rubrum*	• *Prunus pensylvanica* • *A*. *rubrum* • *B*. *lenta*	1.4	6.0
Locust	Harvard Forest	• *P*. *resinosa* • *A*. *rubrum* • *Quercus rubra*	• *A*. *rubrum* • *B*. *papyrifera* • *P*. *pensylvanica*	3.3	6.1
Prescott	Quabbin Reservation Forest	• *Quercus* • *A*. *rubrum* • *B*. *lenta*	• *A*. *rubrum* • *B*. *lenta* • *Q*. *rubra*	0.3	6.7
Prospect	Harvard Forest	• *Picea* • *Prunus serotina* • *A*. *rubrum*	• *P*. *pensylvanica* • *P*. *serotina* • *A*. *rubrum*	6.2	5.8
Ware	Ware River Reservation Forest	• *Pinus* • *Larix* • hardwoods	• *A*. *rubrum* • *P*. *serotina* • *Q*. *rubra* • *Fraxinus americana*	2.1	6.6

Dominant regeneration species reflect the 3 or 4 most abundant tree species pooled across the three treatment plots

Exclosure and control plots were 20 x 20 m in size and located at least 10 m apart from each other. At the start of the experiment, no residual trees were present in the 3 treatments, and virtually all advance regeneration or sprouts were <1m in height; no woody regeneration was >2 m in height. Our tree data were collected in July 2014, 6–7 years after treatment. We established 13 4-m^2^ subplots along 5 successive parallel transect lines within each of the larger 20 x 20 m treatment plots, totaling a 52-m^2^ sampling area (Fig 1C). Three subplots were positioned on the 2 outer and middle rows, and 2 subplots in the second and fourth rows. The center of each subplot was 6 m apart within the same row and 4.5 meters apart between rows. At each of the subplots, all tree and shrub species ≥2 m in height were recorded, and the diameter at breast height (DBH) of each stem was measured. We chose the 2 m height minimum because it corresponded with the upper limit of the herbaceous or forest floor layer [43,44], as well as the predominant browsing zone for eastern North American forest ungulates [45]. Our study therefore focused on the composition, diversity, and density of tree recruitment that was able to successfully pass through the ‘browsing trap’ into the next forest stratum. In a parallel study, we conducted an intensive examination of the herbaceous, low shrub, and small tree seedling flora below 2 m in height [46].

To test for browsing selectivity on different tree species, we conducted an intensive browsing survey in each of the plots in 2012. All woody stems between 0.5 and 2m in height were assessed for past browsing on the leading shoot in each subplot [45, 47]. To monitor animal use of the plots, remote cameras (Reconyx, Inc. (Holmen, Wisconsin) and Cuddeback, Inc. (Greenbay, Wisconsin) were mounted inside each partial exclosure and toward each control plot between 2008 and 2011. Cameras were discontinued after 2011 because the vegetation had grown to a height that effectively blocked the camera’s ability to detect animals. Plots within each block had the same type of camera set to the same delay specifications and were used to calculate the number of deer and moose photographed per week [13]. To avoid multiple counts of the same animal moving in and out of the view of the camera during a single visit, biasing the results, we established a minimum of 5 minutes between photographs of the same animal species to determine a discrete photograph. In addition we sampled pellet groups in April of 2012 in 25, 4m^-2^ circular subplots using the same systematic grid and subplots used for vegetation sampling in 2014, but with an additional 12 subplots.

We used linear mixed effects models (package lmer, the R Foundation for Statistical Computing 2014; R version 2.15.2) with ungulate treatment as fixed effect and block as random effect to determine the response of tree recruitment density, basal area, species richness, and diversity to 3 levels of ungulate browsers. For species richness we examined both species richness (no. species/52 m^2^) and rarefied species richness [48]. For diversity, we used effective number of species defined as exp(Shannon diversity Index) [11,49].

We used either a Gaussian or Gaussian with log link (log normal) distribution for all response variables after examining the residuals to determine the best fit. For hypothesis tests of treatment effects, we used likelihood ratio tests on nested null and treatment models. For significant effects (P ≤ 0.05) of treatment, we performed pairwise comparisons between treatment pairs by simulating the posterior distribution 10,000 times to calculate 95% confidence intervals and approximate P-values for the fixed effects [50,51]. To compare individual species density among treatments, we used Friedman tests with post hoc tests (package agricolae), because the data were frequently heterogeneous. To test for significant differences in community composition among treatments we used adonis (package vegan), the analysis of variance of distance measures (Bray), grouped by block (1,000 permutations)[52]. The stem density for each species was entered into the multivariate test, and rare species that occurred in only 1 of the 18 treatment plots (5.5%) were removed prior to analysis [53].

We examined browsing preferences and overall selectivity by deer in the partial exclosure and by deer + moose in the control plot. We analyzed the 10 most common tree species using IVLEV’s electivity index [54,55]:

$$I_{i=\frac{r_{i}-p_{i}}{r_{i+P_{i}}}}$$

where r = the frequency of browsed stems of a species/the total number of browsed stems of all tree species, and p = the frequency of available stems of a species/the total number of available stems of all tree species. I values range from -1 to 1, with positive values denoting species browsed in greater proportion to their availability and negative values species browsed in lower proportion to their availability. Significant differences in browsed vs. available stems were determined for each species using Chi-squared tests [22].

## Results

Remote cameras detected deer in partial exclosures (0.31 photographs wk^-1^; SE = 0.13) and control plots (0.61 photographs wk^-1^; SE = 0.20; t = 1.15; *P* = 0.32) at each block. Antlered bucks occurred in both partial exclosure and control plots. Deer visits were relatively common in late spring, summer, and fall in both partial exclosure and control plots. Visits were comparatively rare in winter, with deer being detected in early January in both treatments, but absent in February and the first half of March from both treatment plots. Moose were detected only in control plots (0.56 photographs wk^-1^; SE = 0.18). Remote cameras also detected bobcat (*Lynx rufus*), black bear (*Ursus americanus*), coyote (*Canis latrans*), and wild turkey (*Meleagris gallopavo*) inside the partial exclosures, as well as in the control areas, suggesting the partial exclosure plots were permeable to all animal species except for moose. Deer pellet group densities did not differ between partial exclosure (210 groups ha^-1^; SE = 78) and control plots (280 groups ha^-1^; SE = 116; P = 0.35), and moose pellets were only detected in control plots (260 groups ha^-1^; SE = 189).

**Fig 2:**
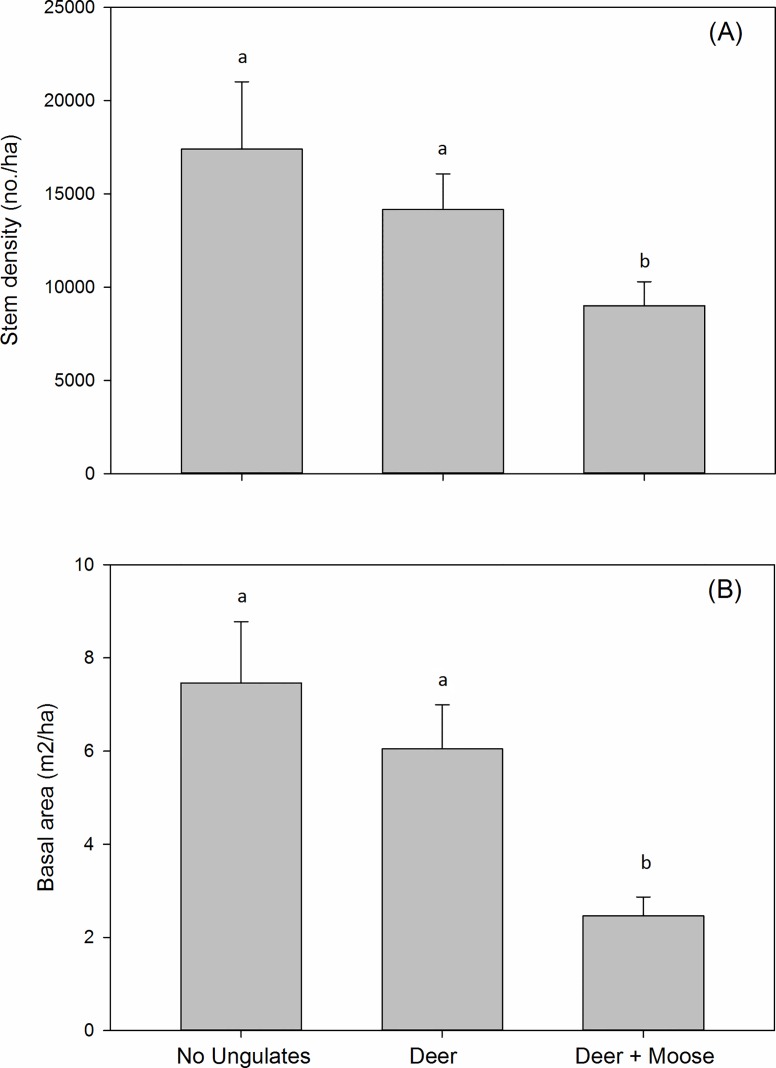
Effects of deer and deer + moose on forest structure. Figure includes (A) woody stem density and (B) basal area for stems ≥ 2 m in height in 6–7 years old patch cut harvests in Massachusetts, USA. Data collected in 2014. Treatment means with the same letter do not differ significantly. N = 6. Bars represent mean ± SE.

Percentage of stems with leading shoot browsed was much higher in deer + moose plots (58%; SE = 8) than in deer plots (16%; SE = 3). Eight of 10 tree species had greater absolute electivity indexes (either positive or negative) in deer plots compared to deer + moose plots (Table 2). *Prunus pensylvanica* was browsed in greater proportion to its availability (χ^2^ = 32.48; df = 1; P<0.0001) in deer plots, whereas *Betula lenta* (χ^2^ = 5.35; df = 1; P = 0.02*)*, *Prunus serotina* (χ^2^ = 4.35; df = 1; P = 0.037) and *Quercus rubra* (χ^2^ = 6.36; df = 1; P = 0.01) were all browsed in lesser proportion to their availability (Table 2). In deer + moose plots *Acer rubrum* (χ^2^ = 14.53; df = 1; P = 0.0001) was browsed in greater proportion to its availability and *Prunus serotina* (χ^2^ = 11.20; df = 1; P = 0.0008) in lower proportion to its availability. Deer + moose reduced stem density (≥2 m in height) by almost half in 2014 relative to ungulate excluded areas (LRT χ^2^ = 8.48; df = 2; *P* = 0.014; Fig 2A). Deer in the partial exclosures caused relatively minor reductions in stem density that did not differ significantly from ungulate exclusion. Effects were similar for basal area, as deer + moose reduced the cross sectional area of stems by 2.5-3-fold relative to no-ungulate and deer plots (LRT χ^2^ = 21.91; df = 2; *P* < 0.0001; Fig 2B). Deer had relatively minor and non-significant effects on basal area in the partial exclosures.

**Table 2:** Browsing selectivity by deer and deer + moose. Data include the 10 most common tree species, and data on each species are pooled across the six blocks in each treatment The Ivlev index (Ivlev 1961) ranges from -1 to 1. Species with positive values were browsed in greater proportion to their availability and those with negative values in lesser proportion to their availability. P values were calculated from Chi-squared tests and reflect differences between proportion of browsed and available stem for each species. Only significant results are shown (P<0.05).

Species	N	Deer	P-value	N	Deer + Moose	P-value
*Acer rubrum*	305	-0.015		430	0.12	<0.001
*Amelanchier* spp.	23	-0.18		23	-0.01	
*Betula lenta*	121	-0.347	0.02	238	-0.03	
*Betula papyrifera*	10	0.09		41	-0.04	
*Fraxinus americana*	7	0.297		68	-0.14	
*Pinus strobus*	17	-1.0		8	-1.0	
*Populus tremuloides*	6	0.16		40	0.03	
*Prunus pensylvanica*	213	0.35	<0.001	115	-0.02	
*Prunus serotina*	65	-0.52	0.04	214	-0.19	<0.001
*Quercus rubra*	128	-0.5	0.01	81	-0.09	

A total of 34 tree and shrub species ≥2 m were recorded in the 18 plots of the six blocks; 92% of the stems sampled were tree species. Community composition did not differ significantly among treatments (Adonis F = 0.57; R^2^ = 0.07; *P* = 0.294), as the same three species–*P*. *pensylvanica*, *A*. *rubrum*, and *B*. *lenta–*dominated the three treatment plots.

**Fig 3:**
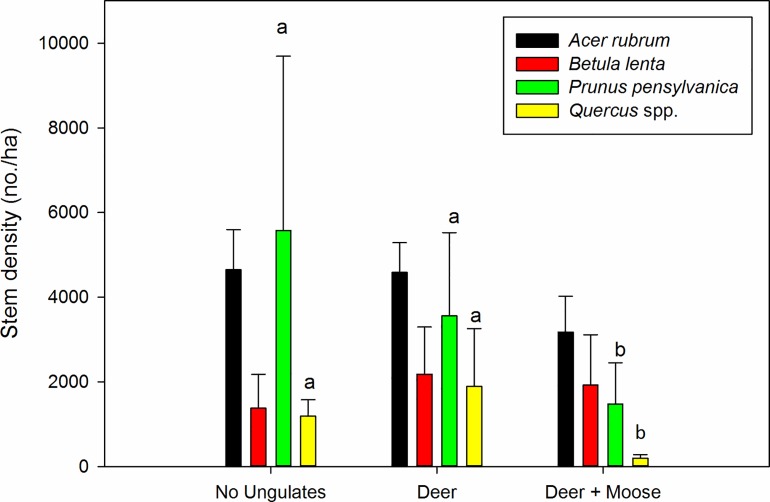
Effect of deer and deer + moose on the density of major tree taxa. Data were collected in 2014 and include stems ≥2 m in height in 6–7 year old patch cuts in Central Massachusetts, USA. *Quercus* spp. = combined *Q*. *rubra*, *Q*. *velutina*, and *Q*. *alba*. Treatment means with different letters differed significantly. Taxa without letters did not differ among treatments. N = 6. Bars represent mean ± SE.

Among individual tree taxa, *P*. *pensylvanica* (Friedman χ^2^ = 8.67; df = 2; *P* = 0.013) and *Quercus* spp. (combined *Q*. *rubra*, *Q*. *alba*, and *Q*. *velutina*];Friedman χ^2^ = 6.64; df = 2; *P* = 0.036) declined by 3.5-fold and 6-fold respectively in deer + moose plots relative to ungulate excluded plots (Fig 3). In the partial exclosures, deer had little or minor effects (Fig 3). *Betula lenta* was the only taxa with a higher density (although non-significant) of stems in the control plot than the other treatments. Neither *A*. *rubrum*, *B*. *lenta*, nor any other tree species differed significantly by treatment, and both species trended upward in terms of relative abundance with the addition of browsers.

**Fig 4:**
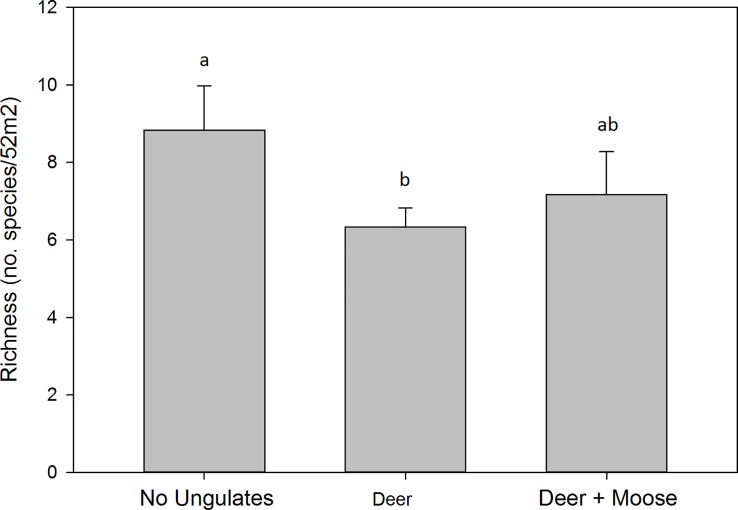
Effect of deer and deer + moose on species richness. Data were collected in 2014 and include stems ≥2 m in height in 6–7 year old patch cuts in Central Massachusetts, USA. Richness differed significantly by treatment. Treatment means with the same letter do not differ significantly. N = 6. Bars represent mean ± SE.

Species richness differed significantly by treatment (χ^2^ = 8.18; df = 2; *P* = 0.017; Fig 4), with richness lower in deer plots by 2.5 species, on average, than in no-ungulate plots (*P* = 0.008). Richness was also marginally lower in deer + moose plots than in no ungulate plots by 1.7 species, on average (*P* = 0.058). Neither species diversity (Friedman χ^2^ = 2.87; df = 2; *P* = 0.238) nor rarefied species richness (χ^2^ = 3.77; df = 2; *P* = 0.15) differed significantly by treatment.

## Discussion

White-tailed deer + moose had strong compounding effects on the structure and individual species, but not diversity, of regenerating forests after 6–7 years of browsing. To our knowledge, this experiment is the first to compare the individual effects of one ungulate browser vs the combined effects of two browsers on tree regeneration in eastern temperate forests of North America. Our results on deer visitation rates (recorded by remote cameras) and pellet group densities did not differ between partial exclosure and control plots (P = 0.31, P = 0.35), although both indices trended lower in the partial exclosures. Although we cannot completely rule out the possibility that the partial exclosure treatment reflected a lower density of deer than ambient deer densities in the control plot, we contend that the evidence for such a difference was weak. Ultimately, the experimental design succeeded in providing two browsing treatments with real world application: low densities of a single herbivore, simulating conditions prior to moose recolonization, vs. low to moderate densities of two herbivores–a novel system after recolonization by moose.

The effects of low densities of deer inside the partial exclosure largely corroborated the existing literature [7,8], as deer in this treatment had relatively minor (and non-significant) effects on stem density, basal area, and species composition. Deer + moose, on the other hand, had strong negative effects on stem density (≥2 m), basal area, and composition demonstrating the extent to which the addition of the larger herbivore foraging alongside the smaller herbivore structured regenerating forest communities. The magnitude of decline in tree recruitment (~50%) by deer + moose, relative to ungulate exclusion, was roughly equivalent to reductions in tree recruitment by 15 deer km^-2^ in 5 year old clearcuts of similar size (1.3–2.6 ha) in Pennsylvania [7]. This very large effect by deer + moose corroborates the prediction that herbivore suppression of woody plants is likely to increase in areas of greater herbivore diversity because the different feeding strategies and reach of the browsers impact a broader range of plant growth stages [3]. Indeed, over time the greater browsing reach of moose likely provided better foraging opportunities for the smaller deer (e.g., by reducing stem heights that afforded a greater density of palatable woody stems and establishing a more open vegetation structure that supported richer herbaceous plant growth) [1,19,46].

The number of moose photographed in the control plots by remote cameras was roughly equivalent to the number of white-tailed deer, suggesting that local moose densities at our sites were probably much higher than densities estimated for the surrounding intact forest [28]. In a recent and nearby study, moose visitation rates recorded by remote cameras were 16 times higher, on average, in logged areas than in nearby undisturbed forest [29]. At the same time, deer used logged forest at a roughly equivalent frequency to undisturbed forest in the aforementioned study [29]. Given that moose are approximately 7 times the mass of deer and are more exclusively browsers [18,32], our remote camera data suggest that moose were the dominant browser species in terms of animal biomass in these patch cut openings.

Sharp reductions in tree recruitment in areas browsed by deer + moose suggest that browsers maintained the vegetation in an earlier successional state than in areas protected from browsing or browsed only by deer [56]. Indeed, plots browsed by deer + moose supported less than half the abundance of herb and shrub species characteristic of interior forest than did no-ungulate and deer plots [46]. A browsing-induced delay in succession could benefit other wildlife species such as shrub and ground nesting birds of conservation concern. Several species prefer areas of low vegetation and few trees, and these species decline in abundance as woody vegetation develops into closed-canopy forest [57]. Still, a delay in succession could simultaneously reduce mid-canopy nesting bird abundance and richness [58].

With respect to tree species composition, deer + moose sharply reduced the dominant pioneer species, *Prunus pensylvanica*, The effect of deer + moose on *P*. *pensylvanica* was comparable to the effect that between 8 and 15 deer km^-2^ had on *P*. *pensylvanica* in other northeastern clearcuts [7,10]. Deer + moose browsing also resulted in a steep decline in *Quercus* recruitment, despite this genus being a less preferred browse taxon than competitors such as *Acer rubrum* (Table 2). Interestingly, the relatively open stand structure and abundant thorny shrubs (*Rubus* spp.)[46]–which are reported to aid *Quercus* recruitment in European temperate forests [2]–in the control plots did not offset the negative effects of herbivory on *Quercus*. Instead, our results corroborated a number of studies showing declines in *Quercus* recruitment with elevated deer densities (≥10 deer km^-2^)[1,8,11].

In contrast to the additive effects of deer + moose on structure and composition, browser effects on species richness did not increase with the addition of moose–that is, deer + moose had a similar effect on species richness as did deer alone (Fig 4). The decline in species richness from deer in the partial exclosure was consistent with other studies that reported declines in richness when deer densities exceeded 4 km^-2^ [10]. Relatively selective browsing by deer alone may have reduced uncommon species below detection levels, resulting in a lower species count. The lack of additional effect on species richness by deer + moose contrasted with many studies reporting greater declines in tree richness and diversity with increasing deer densities and browsing intensities [7,8,10]. Our opposing result could be explained, in part, by a lack of functional redundancy of deer and moose [15]. Different foraging preferences of the two herbivores may have dispersed browsing impacts more evenly across the plant community than the relatively selective browsing of deer alone, thereby reducing plant competition and offsetting the effects of additional browsing intensity on richness [20]. Resource depletion from greater browsing intensity can also lead to reduced selectivity [59]. Additionally, deer + moose browsing reduced the dominant tree canopy layer, which likely provided more growing space for inferior competitors and retained species diversity [9,18]. This pattern was particularly true at one of our sites (Prospect Hill at Harvard Forest) where *P*. *pensylvanica* was extremely dominant and where diversity (effective number of species) was more than twice as high in the control plots as in the full exclosure.

## Conclusions

Low densities of white-tailed deer and moose at the latter species’ southern range limit resulted in dramatic effects on forest structure and composition in disturbed openings–results that were likely influenced by a disproportionately high use of these patch cut openings by moose. At the same time, greater browsing intensity by deer + moose was not accompanied by a greater negative effect on tree species richness relative to deer browsing alone. These results provide additional support that landscapes with two or more herbivores will often have large and complex effects on vegetation [3,20], perhaps due in large part to a lack of functional redundancy among herbivore species [15,60].
